# European Code Against Cancer, 5th edition – a tool for enhancing cancer prevention

**DOI:** 10.1002/1878-0261.70190

**Published:** 2026-01-16

**Authors:** Joachim Schüz, Carolina Espina, Elisabete Weiderpass, Péter Nagy

**Affiliations:** ^1^ International Agency for Research on Cancer (IARC/WHO), World Health Organization Lyon France; ^2^ Department of Molecular Immunology and Toxicology and the National Tumour Biology Laboratory National Institute of Oncology Budapest Hungary; ^3^ Department of Oncology Semmelweis University Budapest Hungary; ^4^ Chemistry Coordination Institute, University of Debrecen Hungary; ^5^ Department of Anatomy and Histology, HUN‐REN–UVMB Laboratory of Redox Biology Research Group University of Veterinary Medicine Budapest Hungary

**Keywords:** cancer policies, cancer prevention, cancer research, European Code Against Cancer, public health

## Abstract

The European Code Against Cancer (ECAC) provides evidence‐based public health recommendations to reduce cancer risk across Europe. First launched in 1987, it is periodically updated mainly to reflect scientific advances. The 5th edition (ECAC5), released for the medical oncology community in 2025 and to be presented to the public in 2026, aims to offer an authoritative and practical tool for cancer prevention for individuals and policymakers. Developed by over 60 experts across five Working Groups, ECAC5 expands the 12 recommendations of the 4th edition to 14, incorporating the latest evidence on modifiable cancer risks and effective medical interventions. Key innovations include new guidance on air pollution, cancer‐related infections, lung cancer screening and strengthened recommendations on tobacco, alcohol, diet, body weight, and occupational and radiation exposures. A major advancement is the addition of dedicated policy recommendations, acknowledging that many prevention measures require supportive environments and regulation. By aligning cancer prevention with broader noncommunicable disease strategies, ECAC5 aims to enhance uptake and impact. Its effective implementation could prevent up to 40% of new cancers in the EU.

## Graphical abstract

The European Code Against Cancer provides evidence‐based public health recommendations to reduce cancer risk across Europe. First launched in 1987, it is periodically updated to reflect scientific advances. The 5th edition aims to offer an authoritative and practical tool for cancer prevention for individuals and policymakers. Its effective implementation could prevent up to 40% of new cancers in the EU.

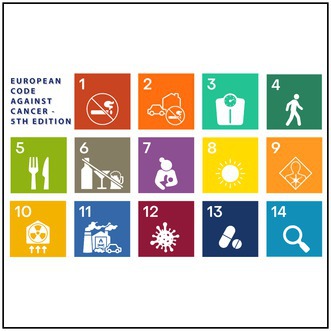

The European Code Against Cancer (ECAC) is a set of public health guidelines aimed at preventing cancer in Europe [1]. It provides evidence‐based recommendations to help reduce the risk of cancer and promote healthier lifestyles and environments. The ECAC initiative originated in 1987 and is being updated over time to reflect new scientific findings and research, with new editions published every 8–10 years. The latest update, the 5th edition of the ECAC (ECAC5), was launched for the medical oncology community in October 2025; it is scheduled to be published in all official languages of the European Union (EU) and will be presented to the general public on World Cancer Day in February 2026 [1]. It has one overarching aim: to provide an authoritative and practical tool for the European population and policy makers to better prevent cancer. The 10 publications included in this Special Issue encompass the scientific justifications and context [2, 3, 4, 5, 6, 7, 8, 9, 10, 11] of the ECAC5.

In comparison with the 4th edition from 2014 [12], the 5th edition represents a substantial improvement of the ECAC due to the following aspects:

1. It is based on the latest scientific evidence on modifiable causes of cancer, as well as on effective medical interventions aimed at reducing the risk of developing or dying from cancer.
2. For the first time, it publishes links with recommendations to policymakers.
3. It aligns the recommendations with those for other noncommunicable diseases for co‐benefits in prevention.
4. It refines the wording of the recommendations to be as equitable and accessible as possible for everyone.

Scientific evidence was systematically assessed by over 60 cancer experts across Europe grouped into five Working Groups who suggested new recommendations or alterations of existing recommendations, with final approval by representatives of the leading cancer prevention institutions and other relevant authorities. This process used the 12 recommendations of the 4th edition as a starting point [12], and resulted in overall 14 recommendations leaving only one of the former recommendations entirely unchanged [1]. Main new elements include recommendations on air pollution [4], expansion on testing, vaccinating against and treatment of cancer‐causing infections [7], and an addition of lung cancer screening [8]. Recommendations on active and passive smoking were expanded and now include e‐cigarettes and similar products [2]. The recommendation on alcohol consumption was strengthened; recommendations on overweight, obesity and healthy diet were updated to include additional products and to better explain the pathway from diet to unhealthy body weight [2, 3]. Recommendations on occupational exposures and radiations were changed mainly for better communication and to make them more actionable [5, 6].

To improve communication of the code [13], a large‐scale evaluation study of over 10 000 participants in eight European countries was carried out. The study indicated that while the length of the recommendations had little effect on recall, providing information on the avoidable risk as a header for each recommendation had a measurable positive impact [10].

Linking the public recommendations with guidance to policymakers is the major advancement of the 5th edition [1]. Importantly, avoidance of cancer‐causing agents or unhealthy choices is not fully under the control of the individual and requires respective frameworks, legislation and support by governments. Furthermore, by publishing the two types of recommendations side‐by‐side, the population becomes more aware of existing policies, which is important because regulations are largely facilitated by the pressure for better protection coming from society. Indeed, the need for implementing policy recommendations was identified by stakeholder consultations [14]. This section was integrated into the World Code against Cancer Framework [15, 16], and initially implemented into the 1st edition of the Latin America and the Caribbean Code Against Cancer [17], with the EU now following this route. Aligning the recommendations better with those for other noncommunicable diseases (such as cardiovascular disease, diabetes or respiratory diseases) provides an added value and makes the Code more attractive for the individual while simultaneously increasing pressure on policymakers [1].

We envision that a ‘better’ ECAC can strongly impact cancer prevention and serve as an important pillar in reducing or slowing down the current 1.3 million cancer deaths in EU27 per year [18]. Following ECAC5 gives individuals, society, policymakers, and the healthcare community a tool at hand, with the strong message that its ideal implementation could prevent up to 40% of new cancers [1]. Efficient utilization of the tool requires the raise of awareness and improvement of its implementation at various levels, for example by its inclusion in national cancer control plans, regional or local prevention campaigns. In addition, barriers to its utilization need to be reduced, including commercial interests and the spread of cancer myths. It is important to emphasize that the ECAC itself deliberately contains recommendations and not commandments, that is despite its many do's and don'ts it is a friendly tool, which is designed to help people avoid premature death and suffering from cancer. The World Code Against Cancer Framework is a flagship programme of the International Agency for Research on Cancer, matching with its mission towards a world where fewer people develop cancer.

## Conflict of interest

None declared. Where authors are identified as personnel of the International Agency for Research on Cancer/World Health Organization, the authors alone are responsible for the views expressed in this article and they do not necessarily represent the decisions, policy or views of the International Agency for Research on Cancer/World Health Organization.

## Author contributions

JS and CE conceived and obtained the funding of the project. CE and JS designed the project. CE was the lead investigator of the conduct of the project. JS wrote the first draft of the manuscript. All other authors commented on previous versions of the manuscript and revised and prepared the final version of the manuscript.
